# Etiology and efficacy of hysteroscopic management in cesarean scar pregnancy: a clinical study

**DOI:** 10.1590/1806-9282.20241912

**Published:** 2025-08-08

**Authors:** Yuhua Zeng, Qiao Luo, Wei Ran, Jiashu Liu, Chengju Zhang, Min Yong

**Affiliations:** 1 The Affiliated Hospital of North Sichuan Medical College, Department of Gynecology – Nanchong, China.; 2 The Affiliated Hospital of North Sichuan Medical College, Department of Reproductive Medicine – Nanchong, China.

**Keywords:** Cesarean scar pregnancy, Risk factors, Hysteroscopy, Effectiveness, Treatment

## Abstract

**OBJECTIVE:**

Cesarean scar pregnancy poses significant risks, including uterine scar rupture and increased postpartum hemorrhage. Effective diagnosis and treatment are critical to improving clinical outcomes. This study aimed to analyze the risk factors for cesarean scar pregnancy and evaluate the efficacy and safety of hysteroscopic diagnosis and treatment, providing a theoretical basis for clinical management.

**METHODS:**

A total of 122 patients with cesarean scar pregnancy, diagnosed via vaginal ultrasound, were included in the study group and treated with hysteroscopic surgery. A total of 90 pregnant women with prior cesarean sections and normal intrauterine pregnancies were selected as the control group.

**RESULTS:**

Logistic regression identified significant cesarean scar pregnancy risk factors: the number of uterine operations, the number of cesarean sections, and a time interval of ≤5 years since the last cesarean (p<0.05). After hysteroscopic treatment, the mean operation time, intraoperative bleeding, and surgical failure rate were 22.31±6.26 min, 57.23±9.12 mL, and 8.03%, respectively. Surgical failure was associated with gestational age, gestational sac position, gestational sac size, diverticulum angle, and blood β-human chorionic gonadotropin levels (p<0.05). Hysteroscopy demonstrated superior diagnostic accuracy over ultrasound for type II and type III cesarean scar pregnancy cases (p<0.05).

**CONCLUSION:**

Hysteroscopic surgery improves treatment outcomes in cesarean scar pregnancy patients. Key risk factors for surgical failure include gestational age, gestational sac position, gestational sac size, diverticulum angle, and blood β-human chorionic gonadotropin levels. Early identification and management of these factors can enhance clinical outcomes and reduce complications.

## INTRODUCTION

Cesarean scar pregnancy (CSP) occurs when a fertilized egg implants at the site of a previous uterine incision, typically in the lower uterine segment. It is a severe long-term complication of cesarean sections and a rare form of ectopic pregnancy. CSP poses significant risks, including uncontrollable massive bleeding, uterine rupture, damage to surrounding organs, and even hysterectomy during or after surgical interventions. These risks gravely threaten women's reproductive health and, in extreme cases, their lives^1^.

Cesarean scar pregnancy is often misdiagnosed as early intrauterine pregnancy due to nonspecific clinical manifestations. This misdiagnosis can result in severe complications, such as massive bleeding during uterine evacuation, making hemostasis challenging. Without timely treatment, ongoing pregnancies can progress to the middle or late stages, leading to life-threatening conditions such as uterine rupture, massive hemorrhage, and placenta accreta. In critical situations, the uterus may need to be entirely removed, severely impacting the patient's fertility and overall health^2^.

The incidence of CSP has risen in recent years due to the increasing prevalence of cesarean sections and improved diagnostic capabilities. Enhanced medical imaging and advancements in clinical techniques have allowed for earlier and more accurate detection of CSP. Understanding the pathogenesis and risk factors of CSP is crucial for clinicians to recognize this condition early and provide effective treatment. Early intervention is essential to minimize complications and improve patient outcomes^3^.

This study aims to investigate the risk factors associated with cesarean scar pregnancy and assess the impact of hysteroscopic diagnosis and treatment on clinical indicators, efficacy, and safety. The findings are intended to provide a theoretical basis for the clinical diagnosis and management of CSP, ultimately guiding better prevention and treatment strategies.

## PATIENTS DATA AND METHODS

### General information

This study included 122 patients with type II/III CSP diagnosed via vaginal ultrasound and hospitalized for surgery in the Department of Gynecology at our hospital from June 2019 to June 2021. Type II: the pregnancy sac (part) was implanted in the scar of the lower anterior uterine wall, and the thickness of the myometrium between the pregnancy sac and the bladder was less than 3 mm; Type III: the pregnancy sac (completely) was implanted in the scar of the lower anterior wall of the uterus, and the muscle layer is convex toward the bladder. The thickness of the uterine muscle between the pregnancy sac and the bladder serosa is obviously ≤3 mm, and the muscle layer is thin or absent. This manuscript conforms to the Enhancing the QUAlity and Transparency Of health Research (EQUATOR) network guidelines.

Inclusion criteria were: (1) history of cesarean section; (2) elevated blood β-human chorionic gonadotropin (hCG) at admission; (3) gestational age ≤12 weeks with no uterine rupture; (4) no surgical contraindications; and (5) desire for fertility with pregnancy planned ≥6 months postsurgery. Exclusion criteria included severe medical complications, liver/kidney dysfunction, hormone-related tumors, mental disorders, organ impairment, or incomplete clinical data. Patients were aged 32.35±5.28 years with a BMI of 21.87±3.17 kg/m^2^. Of these, 70 were classified as type II CSP and 52 as type III CSP. Ninety pregnant women with normal intrauterine pregnancies post-cesarean served as controls. Postoperative follow-up was completed for all patients (100% follow-up rate).

### Research methods

#### Clinical data collection

Clinical data of the subjects were collected, and factors including age, history of induced abortion, history of induced abortion after cesarean section, timing of cesarean section, placental adhesion, and postpartum hemorrhage were analyzed to explore the risk factors of CSP.

#### Diagnosis and treatment of cesarean scar pregnancy patients

Upon admission, all patients underwent chest X-ray, electrocardiogram, liver and kidney function tests, and routine blood examinations. Postoperatively, uterine contractions were promoted, and antibiotics were administered to prevent infection. Hysteroscopy was performed using the OLYMPUS plasma electrotomy system with settings of 120 W for electrocoagulation, 200 W for electrotomy, 350 mL/min water flow, and 100 mmHg uterine pressure. Vaginal endoscopy assessed the cervical canal, uterine cavity, implantation site, vascular distribution, and gestational sac size. Abdominal ultrasound monitored muscular layer thickness and guided the procedure. Lesions were removed through electrotomy, with gestational sac and placental tissue completely excised, ensuring no active bleeding.

Postoperative assessments included operation time, intraoperative bleeding, and surgical failure rates, defined as bleeding >300 mL intraoperatively or >500 mL within 24 h, or complications requiring additional interventions. Clinical data differences between successful and unsuccessful cases were analyzed.

### Statistical methods

In this study, all data were sorted out and corresponding databases were established, and all databases were entered into SPSS 26.0 for data processing. The normal test of measurement data was expressed as (‘x±s), the test between normal multiple groups was F, the independent sample t-test was used for intergroup data, and the paired sample t-test was used for intragroup data. The rate was expressed as % and the test was χ^2^. Multivariate logistic regression analysis was used for influencing factors. When p<0.05, the difference between data was considered to be statistically significant.

## RESULTS

### Univariate analysis of clinical data differences between the two groups

There were differences in the number of uterine operations, the number of cesarean sections, and the time interval between the last cesarean section and CSP between the two groups. The main manifestations were that the number of uterine operations, the number of cesarean sections, and the time interval between the last cesarean section and CSP ≤5 years in the study group significantly increased than those in the control group (all p<0.05), and there were no significant differences in other clinical indicators between the two groups (p>0.05) (Table 1).

**Table 1:** Univariate analysis of the differences in the clinical data between the two groups.

Index		Study group (n=122)	Control group (n=90)	χ^2^/t	p
Age (year)		32.35±5.28	32.09±5.15	0.358	0.721
Body mass index (kg/m^2^)		21.87±3.17	21.56±3.26	0.695	0.488
Number of uterine cavity operations (times)*					
	0	30 (24.59)	34 (37.78)	4.274	0.039
	≥1	92 (75.41)	56 (62.22)		
Times of cesarean section (times)					
	≤1	37 (30.33)	47 (69.67)	10.378	0.001
	>1	85 (52.22)	43 (47.78)		
Time interval between the last cesarean section and CSP (year)					
	≤5	39 (31.97)	54 (60.00)	16.529	<0.001
	>5	83 (68.03)	36 (40.00)		
Uterine position					
	Anterior position	105 (86.07)	83 (92.22)	1.956	0.162
	Posterior position	15 (13.93)	7 (7.78)		

* Including the number of abortions and scraping.

CSP: cesarean scar pregnancy.

### Logistic regression multivariate analysis of the risk factors of cesarean section scar pregnancy

The logistic regression model analysis indicated that the number of uterine operations, the number of cesarean sections, and a time interval of ≤5 years from the last cesarean section to CSP were significant risk factors for scar pregnancy (p<0.05). Specifically, having undergone uterine operations more than once increased the risk (β=0.300, OR 0.351, 95%CI 0.251–0.513, p=0.005). Similarly, patients with multiple cesarean sections had a higher likelihood of CSP (β=0.232, OR 0.261, 95%CI 0.087–0.559, p=0.002). Additionally, a short interval (≤5 years) between the last cesarean section and CSP significantly raised the risk (β=0.524, OR 0.512, 95%CI 0.312–0.755, p=0.001). These findings highlight the critical need for clinical awareness and tailored interventions for individuals with such risk profiles.

### Postoperative operation time, intraoperative bleeding, and surgical failure rate of patients

The average operative time for patients undergoing hysteroscopic treatment was 22.31±6.26 min, with an average intraoperative bleeding volume of 57.23±9.12 mL. The surgical failure rate, defined by criteria such as excessive bleeding or other complications requiring additional interventions, was 8.20%. These findings highlight the efficiency and safety profile of hysteroscopic treatment for cesarean scar pregnancy.

### Comparison of differences between successful and unsuccessful patients

There were significant differences in type, gestational age, gestational sac position, gestational sac size, lower margin, and acute angle of diverticulum between successful and unsuccessful patients (p<0.05). There was no significant difference in other indicators (p>0.05), as shown in Table 2.

**Table 2:** Comparison of the difference between successful and unsuccessful patients.

Index		Successful group (n=114)	Failed group (n=8)	t/χ^2^	P
CSP type (n)					
	Type II	70	0	4.830	0.028
	Type III	44	8		
Gestational week (d)		45.12±11.37	62.63±11.21	-4.214	<0.001
Gestational sac position (n)					
	Upper margin	52	0	6.360	0.012
	Middle margin	47	1		
	Lower margin	15	7		
Gestational sac size (cm)		3.75±0.82	5.42±0.66	-5.626	<0.001
Angle (n)					
	Obtuse angle	50	0	5.945	0.015
	Acute angle	64	8		
hCG (U/L)		33,636.77±1,163.89	71,361.18±1,965.26	-84.190	<0.001

CSP: cesarean scar pregnancy; hCG: β-human chorionic gonadotropin.

### Logistic regression multivariate analysis of influencing factors on the efficacy of cesarean scar pregnancy patients undergoing hysteroscopic surgery

The occurrence of surgical failure is taken as the dependent variable of the study, and the classification, gestational age, gestational sac size, blood hCG, and diverticulum angle are taken as independent variables. The model is selected based on the actual clinical situation. The logistic regression model analysis showed that gestational age, gestational sac size, blood hCG, and diverticulum acute angle were risk factors for surgical failure (p<0.05), as shown in Table 3.

**Table 3:** Logistic regression multivariate analysis of factors influencing the efficacy of cesarean scar pregnancy patients undergoing hysteroscopic surgery.

Index	Results of multivariate analysis					
	β	SE	Wald	OR	95%CI	p-value
Gestational sac position	0.335	1.006	3.157	0.336	0.118–0.591	0.019
Gestational week	1.519	1.035	2.152	4.566	2.600–34.726	0.002
Gestational sac size	0.428	0.536	10.859	0.526	0.371–0.729	0.002
Blood hCG	0.826	0.987	5.293	0.825	0.320–0.963	0.005
Acute angle	0.156	0.135	10.897	0.234	0.081–0.533	0.003

SE: standard error; OR: odds ratio; CI: confidence interval; hCG: β-human chorionic gonadotropin.

### Ultrasound and hysteroscopy for different types of cesarean scar pregnancy ectopic pregnancy diagnosis analysis

Among all diagnosed cases of CSP, there were two cases of type II ectopic pregnancy and three cases of type III. Ultrasound identified four cases of type II and one case of type III, demonstrating a lower diagnostic accuracy compared to hysteroscopy. Hysteroscopy accurately diagnosed all two cases of type II and all three cases of type III ectopic pregnancies. This significant difference in diagnostic accuracy between the two methods (p<0.05) underscores the superiority of hysteroscopy in identifying CSP.

## DISCUSSION

Cesarean scar pregnancy occurs when an embryo implants into the uterine incision scar, typically in the lower uterine segment. CSP is a unique type of ectopic pregnancy and a significant long-term complication of cesarean sections. Its incidence is approximately 1:1,800 pregnancies, accounting for 1.2% of women with a history of cesarean delivery and 6.1% of those with previous cesarean sections among ectopic pregnancies. With the implementation of China's "two-child policy," the rising rate of cesarean sections has led to an increase in CSP cases.

The clinical treatment of CSP includes conservative treatment and surgical treatment, and the treatment plan is individualized, mainly depending on the patient's pregnancy and clinical symptoms. In relevant studies, about 50% of patients chose initial surgical treatment, about 40% chose clinical treatment, and about 10% chose conservative management. There was no difference in complication rates between surgery and medical treatment. Data show that the failure rate of hysteroscopic surgery for CSP is about 5–10%, depending on scar location, size, and physician experience. The failure rate of uterine artery embolization is about 10–15%, and some patients may need a second surgery. To sum up, hysteroscopic surgery is more suitable for patients with fertility needs and small scars. Uterine artery embolization is suitable for patients at high risk of bleeding or for whom hysteroscopic surgery is not suitable. For CSP, early diagnosis is beneficial for patients to avoid surgery through conservative therapy with active monitoring and intramuscular methotrexate therapy. CSP is associated with severe risks such as uterine rupture and placenta accreta, necessitating prompt termination of pregnancy upon diagnosis. Delayed diagnosis and treatment can result in severe complications, including massive bleeding and uterine rupture. If pregnancy progresses to mid or late stages, additional complications such as placental adhesion, implantation, or penetration may arise^4^.

This study identified several risk factors for CSP using the logistic regression analysis. Key factors include the number of uterine surgeries, the number of cesarean sections, and a short interval (≤5 years) between the last cesarean section and the occurrence of CSP (p<0.05). Among these, early symptoms such as threatened abortion may result from a thin scarred muscular layer, decreased contractile force, and insufficient blood supply at the cesarean incision, which collectively increase the risk of CSP. Induced abortion, a common solution for contraceptive failure, is another major contributor. Repeated suction or curettage operations damage the basal endometrium, causing uterine adhesions, endometrial scarring, and poor blood supply. These changes promote embryo implantation in the scarred lower uterine segment, increasing CSP risk. Additionally, the posterior uterine flexion following cesarean sections stretches the lower uterine wall, creating tension and ischemia that can result in scar dehiscence. Elective cesarean sections may also contribute, as poorly formed lower uterine segments during surgery compromise uterine fiber contraction and wound healing^5^.

Evidence-based practices suggest that a double-layer closure of uterine incisions improves healing, reduces bleeding, and prevents local hematoma formation, thereby lowering CSP risk. Placental adhesion, another significant factor, is often associated with multiple abortions and repeated uterine operations, which impair endometrial repair and lead to thinning or adhesions. Effective prevention of intrauterine infection can mitigate the risk of placental adhesion, which is strongly linked to CSP. However, treatments for placental adhesion, such as artificial removal, may cause significant uterine damage and predispose to CSP in subsequent pregnancies^6^.

Advancements in endoscopic technology have positioned hysteroscopy as the gold standard for diagnosing intrauterine conditions. In this study, hysteroscopy demonstrated superior diagnostic accuracy over ultrasound for type II and type III CSP cases. Among two type II and three type III CSP cases, ultrasound misdiagnosed several, whereas hysteroscopy correctly identified all cases (p<0.05). Hysteroscopic electrotomy has become widely used for CSP treatment. Combined hysteroscopic and drug-based treatments for cesarean scar pregnancies have shown significant efficacy in previous studies. In this research, hysteroscopic surgery achieved an average operation time of 22.31±6.26 min, intraoperative bleeding of 57.23±9.12 mL, and a surgical failure rate of 8.03%. Risk factors for surgical failure included gestational age, gestational sac size, blood hCG, and acute angles between the diverticulum and the cervical canal (p<0.05).

Ultrasound imaging often reveals a thin residual muscular layer, defects in the incision, and triangular- or wedge-shaped diverticula, which complicate surgical management. Scar thickness and gestational sac size significantly influence outcomes. Poor elasticity of the fibrous scar tissue and large gestational sacs may impair uterine contractions, increasing the risk of postpartum hemorrhage or excessive oxytocin use. Acute angles between the diverticulum and cervical canal impede visualization of the lesion, while inadequate tension in the anterior wall muscle layer causes blood retention and heightened pressure on the defective layer, affecting surgical success. High serum hCG levels, indicative of vigorous trophoblast growth and strong invasion, also correlate with surgical failure. Pretreatment with methotrexate or similar agents may reduce hCG levels and improve surgical outcomes^7^.

Hysteroscopic surgery has emerged as a reliable, minimally invasive treatment for CSP. Its advantages include a clear surgical field, minimal trauma, reduced blood loss, and faster recovery. Hysteroscopy enables the complete removal of gestational sacs and embryos while reducing serum hCG levels and addressing symptoms like menstrual irregularities. Ultrasound guidance during surgery minimizes risks such as uterine perforation and postoperative complications. Electrocoagulation and hemostasis effectively prevent excessive postoperative vaginal bleeding. Furthermore, hysteroscopy allows precise identification and removal of gestational tissue, reducing the likelihood of recurrent CSP by ensuring comprehensive treatment of vascular implantation sites.

Despite its efficacy, factors influencing surgical outcomes require clinical attention to optimize patient prognosis. Although this study provides valuable insights, there are certain limitations, including the fact that it was a single-center study and there may be potential selection bias. In addition, there is a lack of long-term fertility outcomes for patients after hysteroscopy. In the future, multicenter studies and prospective univariate studies can be conducted to further evaluate the safety and efficacy of hysteroscopic surgery for CSP, long-term fertility outcomes were observed through follow-up, and finally provide a reference for the treatment strategy of CSP.

## CONCLUSION

This study identified key risk factors for type II/III cesarean scar pregnancy, including the number of uterine operations, multiple cesarean sections, and a short interval (≤5 years) between the last cesarean section and CSP. Hysteroscopic surgery demonstrated significant efficacy, with reduced operation time, minimal bleeding, and a low failure rate. Critical factors influencing surgical outcomes include gestational sac position, gestational sac size, diverticulum angle, and blood hCG levels. Combined with the results of this study, the effect of hysteroscopic surgery in the treatment of CSP will be affected by a variety of factors, which can provide a strong basis for the management of CSP patients and the selection of treatment plans. In clinical practice, more appropriate treatment plans can be selected according to the actual situation of patients, so as to improve the diagnosis and treatment level of CSP.

## Data Availability

The datasets generated and/or analyzed during the current study are available from the corresponding author upon reasonable request.
